# Characterization of thermo/halo stable cellulase produced from halophilic *Virgibacillus salarius* BM-02 using non-pretreated biomass

**DOI:** 10.1007/s11274-022-03446-7

**Published:** 2022-11-24

**Authors:** Naeima M. H. Yousef, Asmaa M. M. Mawad

**Affiliations:** Botany and Microbiology Department, Faculty of Science, Assiut University, Assiut, 71516 Egypt

**Keywords:** Agricultural wastes, Cellulase, Halostable, Lignocellulosic, Thermostable, *Virgibacillus* sp.

## Abstract

The production of extremozymes from halophilic bacteria has increased significantly due to their stability and efficiency in catalyzing a reaction, as well as their capacity to display optimum activity at various salt concentrations. In the current study, the halophilic bacterium *Virgibacillus salarius* strain BM-02 could utilize many non-pretreated substrates including cellulose, corn stover, sugarcane bagasse and wheat bran as a sole carbon source. However, wheat bran was the best substrate for achieving optimum saccharification yield (90.1%). The partially purified cellulase was active and stable at a wide range of pH (5–8) with residual activities > 58%. Moreover, it was stable at 5–12% of NaCl. Metal ions have a variable impact on the activity of partially purified cellulase however, Fe^+3^ exhibited the highest increase in the cellulase activity. The enzyme exhibited a thermal stability at 40, 50 and 60 °C with half-lives of 1049.50, 168.14 and 163.5 min, respectively. The value of V_max_ was 22.27 U/mL while Km was 2.1 mM. The activation energy of denaturation E_d_ 69.81 kJ/mol, the enthalpy values (ΔH_d_) were positive, and the entropy values (ΔS) were negative. Therefore, *V. Salarius* is recommended as a novel promising halophilic extremozyme producer and agricultural waste remover in the bio-industrial applications.

**Graphical abstract:**

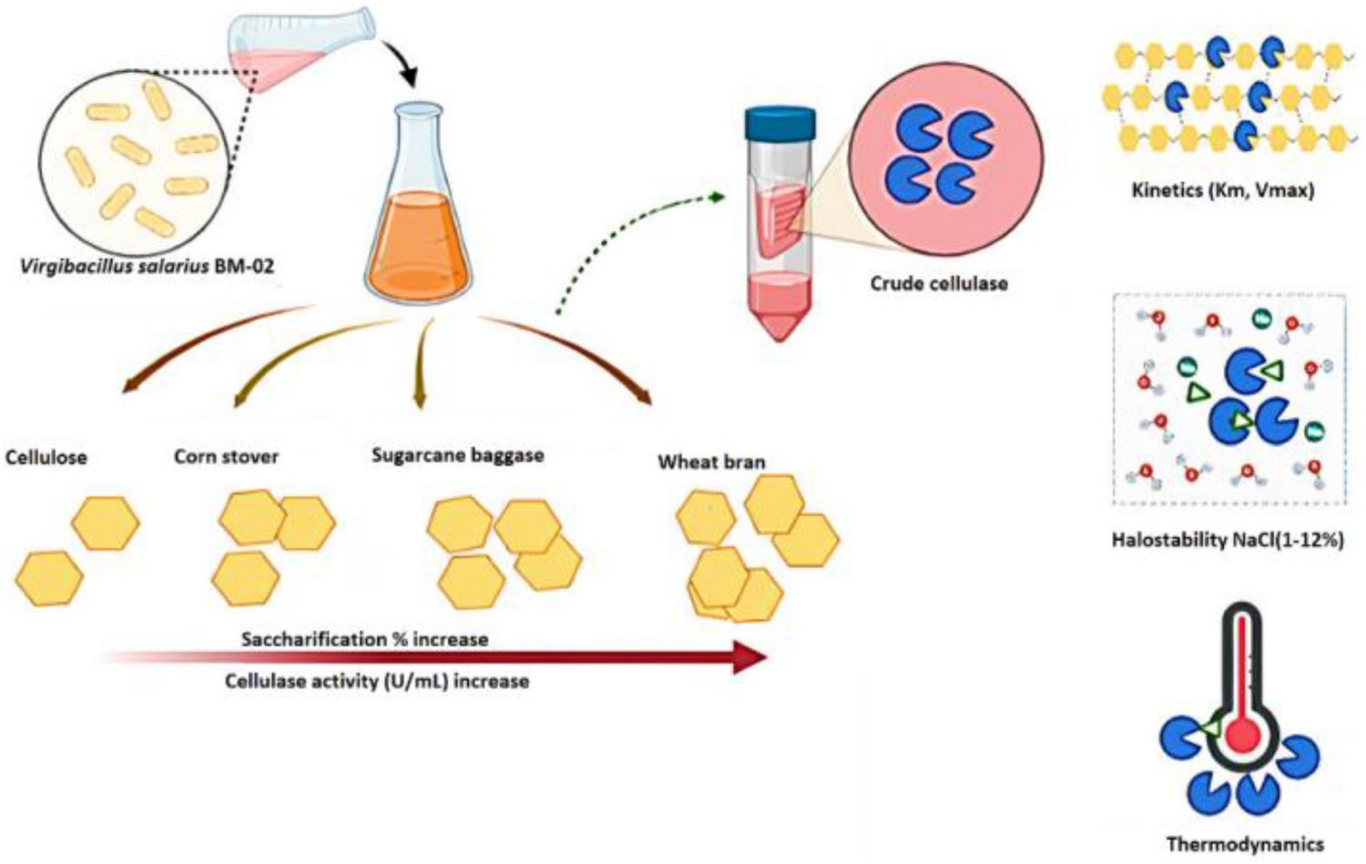

## Introduction

Elimination of lignocellulosic materials from the environment is an urgent need to solving many global problems such as energy requirements, resources shortages, environmental pollution, and food safety. Many studies have been conducted to establish efficient strategies for converting cellulosic and lignocellulosic biomass into high-valuble compounds (Harnvoravongchai et al. 2020; Ximenes et al. 2021). However, the persistence nature of lignocellulosic biomass has posed a significant barrier to cellulosic biomass conversion and exploitation. Chemical treatments of lignocelluloses using solvents, acid and/or base has many restrictions as they have high operation pressure, high cost, low glucose yield and production of toxic byproducts such as furfural (Maurya et al. 2015). Enzymatic (biological) hydrolysis is representing an efficient strategy for removal of lignocellulose due to its mild operation condition, cost effectiveness, high specificity, and selectivity and environmentally ecofriendly (Karim et al. 2021; Menon and Rao 2012; Salah et al. 2021; Yousef et al. 2020).

Cellulase is a key enzyme that play a crucial role in plant biomass degradation (Mani-López et al. 2012). It is a group of enzymes that catalyze the breakdown of β-1,4 glycosidic bond of cellulose molecule. Therefore, cellulases are classified based upon the specific hydrolytic site into three groups: (i) endoglucanase; (ii) exoglucanase and (iii) β-glucosidase (Mosier et al. 1999). Cellobiohydrolase, endoglucanase or carboxymethylcellulase (CMCase), and beta-glucosidases, in combination, are required for complete hydrolysis of the cellulosic matters (Mosier et al. 1999). Despite an enormous consumption of natural cellulosic materials, there are still a lot of waste products and cellulose-containing raw materials are not exploited. However, developing techniques that are commercially advantageous is a challenge in this regard.

Cellulase enzymes have been extracted from different biological sources such as *Cellulomonas* sp (Poulsen et al. 2016; Saratale et al. 2010)*, Pseudomonas* sp (Cheng and Chang 2011), *Bacillus,* and *Micrococcus* (Sethi et al. 2013). However, the industrial applications of these enzymes are faced many struggles like moderated functional temperature range, mild pH values, low salts, response to metal ions and low durability and stability under industrial settings (Iyer and Ananthanarayan 2008). Therefore, tremendous research effort has been continuously stablished to seek for novel thermotolerant biomass catalyzing enzymes from different environment.

Extremophilic bacteria are important in a biorefinery because they provide unique metabolic pathways as well as catalytically stable enzymes. that can serve as biocatalysts under severe industrial circumstances (Zhu et al. 2020). Extremophilic bacteria could thrive under harsh environmental conditions such as low/ high temperature, acidic/alkaline pH values, salinity, and pressure that struggles the survival of most living organisms (Singh et al. 2019). s. Many literatures focused on these types of bacteria in order to understanding their metabolic cycles during industrial applications because of their good thermal stability, favorable substrate solubility, rapid mass transfer rate, and reduced pollution risk during industrial setting operations (Chen and Jiang 2018; Singh et al. 2019). *Virgibacillus* species was halophilic bacterial species that were involved in applications such as protease, amylase, bioflocculant and exopolysaccharide production (Cosa et al. 2011; Gomaa and Yousef 2020; Sinsuwan et al. 2007).

Therefore, the main objective of this investigation to determine the optimum operation conditions of cellulase enzyme that extracted from halophilic *V. salarius* strain BM-02. In addition to study the kinetics and thermodynamics of the partially purified enzyme.

## Materials and methods

### Bacterial strain

The halophilic bacterial strain *Virgibacillus salarius* BM02 in the current study was previously isolated from a bottom sediment sample that was collected from Wadi El-Natron, Egypt (Gomaa and Yousef 2020).

### Substrate

Wheat bran is a cellulosic waste that used in this study as a main substrate for induction of cellulase enzyme. A weigh of 1000 g of untreated wheat flour was milled through 1-mm mesh screen to separate wheat bran. A hundred gram of produced wheat bran was then used for chemical analysis. A volume of 40 mL methanol was used to extract 0.5 g of each milling fraction for 20 h. Then the centrifugation was performed at 3000 g for 20 min, the produced supernatant was evaporated, and the residue was solubilized in methanol to a final volume of 2 mL. Total carbohydrate, protein, fibers, ash and minerals were estimated by the method described by (Rasper and Walker 2000).

*Total polyphenol content* of wheat extracts was measured by the method of (Singleton et al. 1965) using Folin-Ciocalteu reagent. A volume of 100 µL of each the sample extract was mixed with an equal volume of the Folin-Ciocalteu reagent and 1 mL of 20% (w/v) NaCO_3_. The mixture was centrifuged at 11,000 × *g* (Eppendorf MiniSpin, Germany) for 10 min. The supernatant was used for determining the total polyphenol content at 690 nm using a BioTek Microplate Reader (ELx800, USA). Gallic acid (25–100 mg L^–1^; R^2^ = 0.999) was used as the standard.

*Total flavonoid content* was determined using the modified method of (Fattahi et al. 2014). A volume of 100 µL of wheat extract was added to 20 µL of methanolic solution of aluminium chloride 5% (w/v) and centrifuged (Eppendorf MiniSpin, Germany) for 10 min at 11,000 g. The supernatant was used for determining the total flavonoid content at 405 nm. Quercetin (2–20 mg L^–1^; R^2^ = 0.998) was served as a standard.

### Determination cellulolytic capability of *V. salarius* BM-02

The bacterial culture was grown on Luria–Bertani broth (LB) medium supplemented with 3% NaCl for 24 h at 37 °C and 120 rpm in an orbital shaker. The bacterial cells were harvested by centrifugation at 5000 rpm for 10 min. The pellet was washed twice and resuspended in phosphate buffered saline (PBS). A volume of bacterial suspension (OD600 = 0.3) was inoculated into a Bushnell Haas (BH) broth medium with a composition (g / L): NH_4_NO_3_ (2.5); KH_2_PO_4_ (2.0); K_2_HPO_4_ (1.0); MgSO_4._ 7H_2_O (0.2); NaCl (0.2); CaCl_2_ 6H_2_O (0.02), FeCl_3_·6H_2_O (0.05) pH 7.0. The medium was supplemented with 20 g/L of wheat bran as the sole carbon source to stimulate induction of cellulolytic enzyme. Then it was incubated at 37 °C and 120 rpm in an orbital shaker for 6 days.

### Determination of cellulase activity

An aliquot of 5 mL of bacterial growing in BH was aseptically withdrawn each day interval. The bacterial culture was filtered using filter paper, then the filtrate was centrifuged at 5000 rpm for 10 min to get rid of wheat bran debris and bacterial cells, respectively. The clear supernatant contained of cell free enzyme extract.

Cellulase activity was assessed by mixing 900 µL of 1% carboxymethyl cellulose (CMC) dissolved in sodium citrate buffer (50 mM, pH 6.5) (w/v) with 100 µL of crude enzyme extract; the reaction mixture was incubated at 40 °C for 30 min boiled for 10 min (Miller 1959). The reaction was terminated by adding 1.0 mL of 3, 5-dinitrosalicylic acid (DNS). The developing color was determined after cooling by measuring the absorbance at 540 nm using spectrophotometer. By using glucose as a reference, the free reducing sugars were measured. Under the assay circumstances, one unit of cellulase is defined as the quantity of enzyme required to release one mole of glucose equivalent (reducing sugar) per minute (Wood and Bhat 1988).

### Substrate utilization and agricultural wastes saccharification

The susceptibility of *V. salarius* BM02 to utilize agricultural wastes as a main substrate for cellulase production and as carbon and energy sources was assessed. A volume of 50 mL of BH broth medium was inoculated with an overnight with LB growing *V. salarius* BM02 (OD600 = 0.3) after centrifugation and twice washing. Then the medium was separately supplemented with 2% of either cellulose, corn stover, sugarcane bagasse or wheat bran under the same previously mentioned incubation condition for 72 h.

Crude cellulase activity (U/mL) was determined as the procedures mentioned before while the quantity of reducing sugars liberated by the enzymatic hydrolysis was determined according to the method of DNS. The tubes were transferred in a boiling water bath for 10 min, A volume of 1.0 mL of sample was mixed with 2.0 mL of DNS reagent and the absorbance of each sample was determined at 540 nm after cooling (Hu et al. 2008). The amount of the reducing sugar released (mg/ml) was measured by using a calibration curve of glucose (1–100 mg/mL). The saccharification percentage was estimated by applying equation as follows: (Alrumman 2016; Srivastava et al. 2021)

1 $${\text{Saccharification}}\,\left( \% \right)\, = \,\left[ {{\text{reduced}}\,{\text{sugars}}\,\left( {{\text{mg}}/{\text{mL}}} \right)\, \times \,0.9/{\text{initial}}\,{\text{substrate}}\,{\text{conc}}.\left( {{\text{mg}}/{\text{mL}}} \right)} \right]\, \times \,100,$$

where 0.9 was the factor used to convert polysaccharide to monosaccharide accounting for water uptake during hydrolysis.

### Determination the optimum concentration of the best agricultural waste

Based upon the previous test, the best agriculture waste that stimulated the highest cellulase productivity was selected to detect the optimum concentration of it that induced high cellulase productivity. The sterilized BH  media were inoculated with the bacterial cells (OD600 = 0.3). Then different concentrations of the selected agriculture waste (10, 20, 30*,* 40 and 50 g/L) were separately supplemented to the media and incubated at 30 °C for 72 h in a rotatory shaker at 120 rpm.

### Partial purification of cellulase

Purification was performed on the cell free crude enzymes extract obtained from 72-h growing *V. salarius* BM02 under the optimum conditions. The crude culture was filtrated, the filtrate was centrifuged at 10,000 rpm for 10 min to remove the cells and remaining media. The supernatant was overnight precipitated with saturated (NH_4_)_2_SO4, and the pellet was recovered by centrifugation at 12,000 rpm for 10 min. The pellet was resuspended in 100 mM phosphate buffer, pH 7.0, and dialyzed overnight at 4 °C against 10 mM phosphate buffer. To achieve high purification, buffer was altered at each 1 h interval (Li and Yu, 2013). The dialysate was preserved at − 20 °C for further use. The cellulase activity was determined as previously mentioned procedure.

The total protein content (µg/mL) was assessed according (Lowry et al. 1951). The protein concentration was estimated using bovine serum albumin as a standard. The OD660 of the reaction mixture was determined.

### Characterization of partially purified cellulase

#### Effect of pH, temperature, salinity on cellulase activity and stability

To determine the impact of pH and temperature on stability and activity of the partially purified cellulase, the enzymatic reactions were carried out by mixing an equal volume of cellulase and 100 mM citrate buffer, pH 6.5, supplemented with 1% CMC. For determine the effect of pH, the cellulase was incubated with a substrate in a series of buffers with different pH systems (50 mM) as follows: (i) pH 4.0–6.5 using citrate buffer; (ii) pH 7.0–8.0 using phosphate buffer; and (iii) pH 9.0 using glycine/NaOH for 60 min. Furthermore, the impact of temperature was assessed by allowing the reaction mixture to perform at different temperatures from 40 to 70 °C for 30 min under optimal pH value. The impact of salinity was performed at optimum pH and temperature by adding serial concentration of NaCl (1–12%) to the reaction buffer. The hydrolytic enzyme activities were measured under standard test circumstances.

To evaluate the pH, thermo and halo-stabilities of partially purified enzyme, the enzyme reaction mixture was incubated at optimum conditions of each parameter, i.e., pH level, temperature degree and NaCl concentration for an hour, and residual hydrolytic activity was determined using DNS method as described above.

#### Impact of some additives on the enzyme activity

The impact of metal ions on the cellulase enzyme production was assessed by separately incubating 5 µM of CuSO_4_, MgSO_4_, CaCl_2_, FeSO_4_, MnCl_2_, ZnSO_4_ or NH_4_CL_2_ with enzyme reaction mixture for 30 min under optimal assay condition. Other additives such as Ethylenediaminetetraacetic acid (EDTA) (5 mM) and surfactant like ٍSodium Dodecyl sulfate (SDS) 1% were separately tested. The cellulase activity was determined as the method described above where the activity without any addition of metal ion was serve as control. The rate of increase or decrease in the cellulase activity was calculated from the following equation (Wang et al. 2018):

2 $${\text{Cellulase}}\,{\text{activity}}\,{\text{increase}}\,{\text{rate}}\,\% \, = \,100*\,\left( {{\text{activity with metal ion}}\, - \,{\text{activity of control/activity of control}}} \right).$$

#### Cellulase-substrate kinetics

The influence of substrate concentration on the kinetics of enzyme–substrate reactions was examined using different concentrations of CMC (1–10 mg/mL) under the optimum assay conditions of partially purified cellulase. The kinetics parameters were used to calculate km and v_max_ from Lineweaver–Burk plot. Michaelis–Menten Eq. (3) was used to plot a straight-line of V^*−*1^ against S^*−*1^ as follow:

3 $$1/{\text{V}}\, = \,{\text{K}}_{{\text{m}}} /{\text{V}}_{{{\text{max}}}} \, \times \,1/\left[ {\text{S}} \right] + 1/{\text{V}}_{\max } ,$$

where, km Michaelisian-constant (g/L), S; the substrate concentration (g/L), V is the starting rate (g L^−1^ min^−1^) and maximum velocity (Vmax) values of the enzymes were estimated from the slope and intercept of the straight Lineweaver–Burk plot.

#### Impact of cellulase concentration

The concentration of enzyme in the reaction mixture was determined by separately applying of different volume of enzyme extract (25–200 µL/mL of reaction mixture). Then the reaction mixture was incubated at optimum pH, temp, and NaCl conditions for 30 min. After boiling for 10 min, the process was halted by adding 1.0 mL of DNS. A spectrophotometer was used to determine the developing color at 540 nm.

#### Thermodynamics during thermal inactivation of cellulase

The values of cellulase residual activity at each temperature were used to calculate the thermal inactivation rate constant (K_d_/min) from the slope of the curves in the first order plot of ln (% residual activity, RA) versus time (t, min) according to the following equation

4 $${\text{In}}\,\left( {{\text{RA}}} \right)\, = \, - {\text{K}}_{{\text{d}}} \,{\text{t,}}$$

The half-life period of cellulase (t _1/2_, time where residual activity reaches 50%) was calculated as:

5 $${\text{t}}_{1/2} \, = \,{\text{In}}\,\left( 2 \right)/{\text{K}}_{{\text{d}}} ,$$

The decimal reduction time (D-value) is the time needed to sustain 10% RA was calculated as: -value (the temperature necessary to decrease the D-value by a logarithmic cycle) was calculated using the equation from the slope of the graph plot of log (D) versus temperature (°C) as follow:6

The thermodynamic parameters: Gibbs free energy (ΔG_d_), entropy (ΔS_d_) and enthalpy (ΔH_d_) of activation of the thermal denaturation of cellulase can be approximated using the rearrangement equation, which expresses the temperature dependency on the deactivation rate constant as follow:

7 $${\text{Ln}}\,\left( {{\text{K}}_{{\text{d}}} /{\text{T}}} \right)\, = \,{\text{Ln}}\,\left( {{\text{k}}/{\text{h}}} \right)\, + \,\left( {\Delta {\text{S}}_{{\text{d}}} /{\text{R}}} \right)\, - \,\left( {\Delta {\text{H}}_{{\text{d}}} /{\text{RT}}} \right),$$

where kd is the deactivation rate constant (min ^−1^), T is the absolute temperature (K), k is the Boltzmann constant (1.3806 × 10^–23^ J K-1), h is the Planck’s constant (2.3854 × 10^–30^ J h), R is the gas constant (8.314 × 10^–3^ kJ mole -1 K-1), ΔS_d_ is the variation in entropy (kJ mole-1 K-1) and ΔH_d_ is the variation in enthalpy (kJ mole-1).

From Eq. (7), The ΔH_d_ and ΔS_d_ can be approximated from the slope and intercept of the plot of ln(k_d_/T) versus (1/T),  respectively. The alter in Gibbs free energy ΔG_d_ of the cellulase can be calculated by applying the following formula:

8 $$\Delta {\text{G}}_{{\text{d}}} \, = \,\Delta {\text{H}}_{{\text{d}}} \, - \,{\text{T}}\Delta {\text{S}}_{{\text{d}}} .$$

### Statistical analysis

All experiments were performed by using triplicate experimental sets. Results were presented as the mean ± SD. One-way ANOVA using graph-pad Prism software.

## Results

### Chemical analysis of wheat bran

The chemical constituents of wheat bran used in this study were investigated**.** It predominantly comprised total carbohydrate (64%), protein (12%), polyphenols (8.17%), flavonoids (0.2%), ash (4%), fibers (8.8%), Mg++ ion (0.24%) and sulfate ion (0.09%).

### Cellulolytic capability of V. salarius BM02

**Fig. 1:**
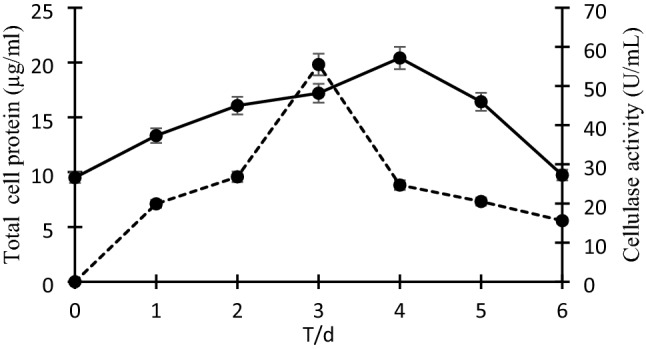
The correlation between the total cellular protein (µg/mL); (solid line) and the induced cellulase activity (U/mL); (dotted line) along with the time (day). Error bars represent the standard deviation (SD ±) of three replications

In the current study, the total protein concentration was estimated for 6 days (24 h interval) of incubation (Fig. 1). The results illustrated that the maximum induction of cellulase enzyme (55.5 u/mL) was detected at 72 h of incubation while the exponential protein concentration (20.41 µg/mL) was detected at 96 h. After these times, the depletion in the bacterial growth (protein) and the cellulase activity was significant (*P* ≤ *0.05*) detected.

### Substrate utilization and saccharification yield

**Fig. 2:**
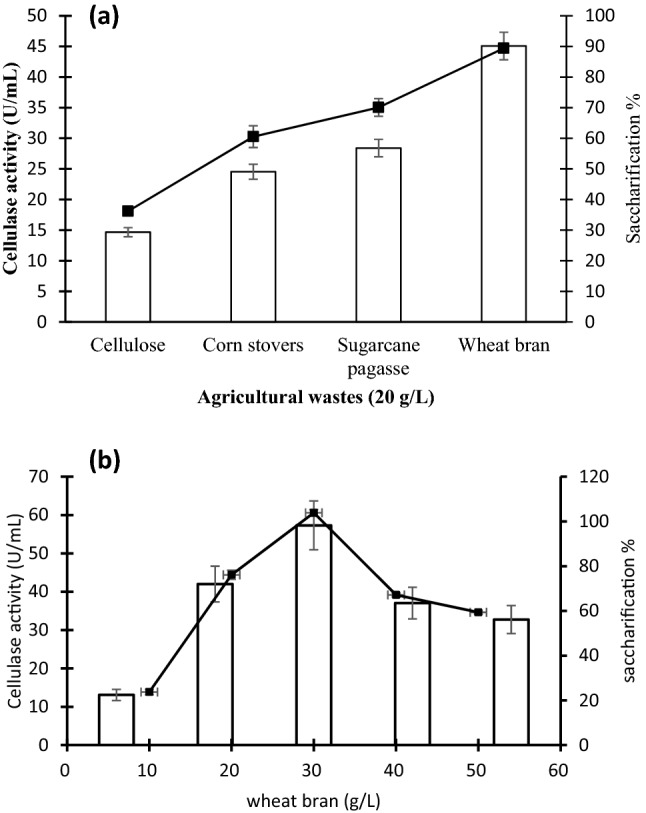
**(a)** The impact of different types of agricultural wastes (20 g/L) and (**b):** the impact of wheat bran concentration (optimum substrate) on the cellulase activity (U/mL); (line) and the percentage of saccharification (column). Error bars represent the standard deviation (SD ±) of three replications. ^**^ significant difference (P ≤ 0.05)

The ability of *V. salaries* *BM-02* to degrade agricultural wastes, cellulase activity and the percentage of saccharification was determines as shown in (Fig. 2a). The results showed that the saccharification percentage that was liberated during cellulose (29.32%) utilization was significantly (*P* ≤ *0.05*) low compared with corn stover (49.07%) and sugarcane bagasse (56.8%) utilization. However, the highest (*P* ≤ *0.05*) saccharification percentage (80.8.1%) was liberated during wheat brane utilization.

### Cellulase-substrate specificity

Regards to the cellulase activity on different substrates, the results lined with the saccharification percentage as shown in Fig. 2a. It was noticed that the cellulase that was extracted from *V. salarius BM02* was active towards cellulose, and agriculture wastes such as corn stover, sugar bagasse and wheat bran. However, the lowest (*p* ≤ *0.05*) cellulase activity was detected during consumption of cellulose as a carbon source of (18.1 U/mL) while the highest (*p* ≤ *0.05*) cellulase activity (44.7 U/mL) value was detected during wheat bran utilization. Moreover, the activity of enzyme towards many substrates indicating that the cellulase induced from *V. salarius* BM02 was a wide substrate specificity.

### Determination of the optimum substrate concentration

Based upon the previous experiment, the optimum substrate that enhanced cellulase induction and yeilded the highest saccharification percentage was wheat bran. So, various concentrations of wheat bran (10–60 g/L) were assessed to determine the optimum concentration of the substrate . Results showed that 30 g/L was the optimum concentration that induced the highest saccharification (95.2%) and the highest cellulase activity (60.2 U/mL) compared with other concentrations after incubation at 30 °C for 72 h (Fig. 2b).

### Effect of pH and salinity on cellulase activity and stability

**Fig. 3:**
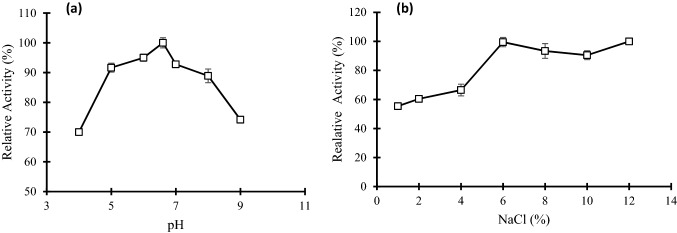
The impact of pH (**a**), and NaCl concentration (**b**) on the relative activity of partially purified cellulase. Error bars represent the standard deviation (SD ±) of three replications

The impact of pH on cellulase activity was determined at different pH values ranged from 4 to 9 (Fig. 3a). Cellulase exhibited activity on all selected pH ranges however, the optimum pH was found to be (pH 6.5). Above the optimum pH, the activity slowly decreased to pH 8.0 after that level the activity sharply dropped at pH 9.0. Cellulase activity was quite stable throughout a wide pH range, with more than 74% residual activity after a 60 min preincubation in the pH range 5.0–9.0. Cellulase stability was gradually increased with increasing pH values and the optimal pH for maximum cellulase activity was found at pH 6.5. More than 90% of the initial activity remained at pH 5.5 and 7.0.

### Impact of NaCl on the activity and halo stability of cellulase

The bacterium *V. salarius* BM02 was able to grow and produce cellulase up to 12% of NaCl concentration which confirming the halophilic nature of the bacterium. The partially purified cellulase enzyme that extracted from bacteria was active in the range of 1–12% salinity. However, the maximum activity was determined at concentration range 6- 12% (Fig. 3b).

### Effect of additives on the cellulase activity

In the current investigation, the incubation of enzyme reaction with metal ions showed a noticeable (p ≤ 0.05) increase in the cellulase activity compared to mixture free ions (control) (Table 1). The cellulase activity that was incubated with Fe^+3^ ions exhibited highest rate (120%) (p ≤ 0.05) compared with other metal ions. No significant variation has been detected between the activities rate of Zn^+2^ (33.6%) and Mg^+2^ (32.9%) or Cu^+2^ (12.33%) and Mn^+2^ (10.2%). The Ca^+2^ and NH_4_^+^ ions showed the lowest (p ≤ 0.05) increase in the cellulase rate 6.9% and 5.5%, respectively as demonstrated in (Table 1). Regarding to the impact of EDTA and SDS, they both exhibited inhibitory impact on the enzyme activity (Table 1).

**Table 1:** The impact of metal ion concentration on the partial purified cellulase activity increase rate %

Metal ions	Enzyme activity increase rate %
Cu^+2^	12.33 ± 0.3
Mg^+2^	32.9 ± 1.1
Ca^+2^	6.9 ± 1.7
Fe^+3^	120.6 ± 2.1
Mn^+2^	10.2 ± 1.4
Zn^+2^	33.6 ± 1.8
NH^+^	5.5 ± 0.9
EDTA	− 59 ± 0.9
SDS	− 89 ± 1.01

The rate was compared to control (reaction mixture with no addition of metal ion)

### Cellulase kinetics

**Fig. 4:**
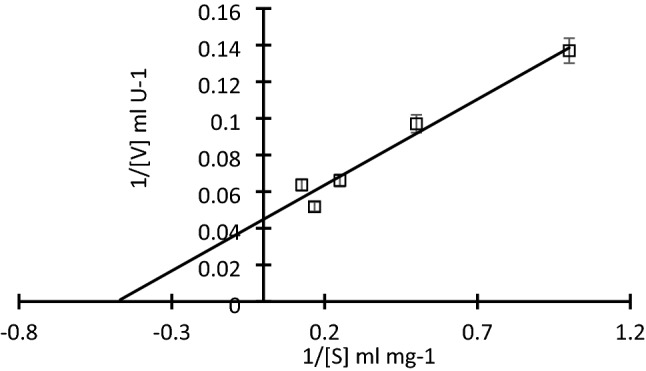
The Lineweaver –Burk plot to estimate Km, app and Vmax of cellulase produced by *V. salaries* under optimum conditions, Error bars represent the standard deviation (SD ±) of three replications

The partialy purified enzyme's substrate specificity was determined by testing its activity in the reaction mixture against various concentrations of CMC solution (1–10 mg/ml). According to the Lineweaver–Burk plot (Fig. 4), the maximum value of enzymatic velocity (Vmax) was 22.27 U/mL. Theoretically, the value of Michaelisian constant km is defined as the affinity of substrate towards the enzyme. The value of km is inversely proportional to the affinity of enzyme and substrate. Km was thus calculated to be 2.1 mM.

### Thermodynamics

**Fig. 5:**
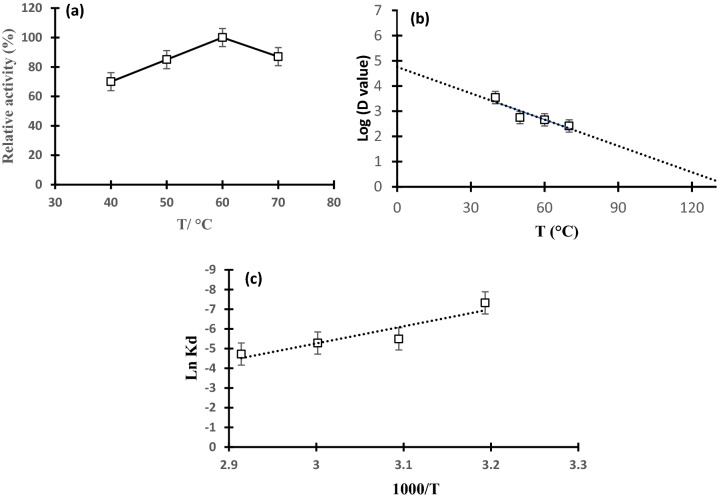
The impact of temperature on the relative activity of partially purified cellulase (**a)**, D value-plot to calculate  -value (the temperature required to reduce the D-value by one logarithmic cycle) (**b)** and First-order Arrhenius plot for determination of activation energy of denaturation (Ed) of cellulase under the optimum enzyme conditions (**c).**

The impact of temperature on the activity and stability of cellulase was illustrated in Fig. 5a, It was noticed that cellulase was thermo-active at high temperature. It was noticed that cellulase was active at temperature range from 40 to 70 °C, however, the maximum activity (100%) was determined at 60 °C. in addition to, it was observed that, the deactivation rate constant increased with an increase in the temperature. For instance, the rate of deactivation constant was 6.6 × 10^–4^, 4.12 × 10^–3^, 0.0051 and 0.0089 min at 40 °C, 50 °C, 60 °C and 70 °C, respectively (Table 2).

**Table 2:** Inactivation kinetics and thermodynamics parameters for thermal inactivation of cellulase

T (^°^C)	K_d_ (min)	*t*_1/2 (min)_	D-value	*ΔH* (KJ/mol)	*ΔG*(KJ/mol)	*ΔS* (J/mol/K)
40	0.00066	1049.50	3486.36	69.57	106.5	− 117.97
50	0.004122	168.14	558.54	69.49	101.73	− 99.82
60	0.005078	136.50	453.45	69.41	101.19	− 95.45
70	0.008902	77.86	258.65	69.33	99.73	− 88.65

Kd thermal inactivation rate constant, R2 coefficient of correlation, t1/2 half-life, D-value decimal reduction time, Ed activation energy of denaturation, ΔH enthalpy, ΔG free energy, ΔS entropy. Ed = 69.81 kJ/mol

The half-life period of an enzymes (t _½_) is an important parameter that determined for how long the enzyme will withstand during the operation process at high temperature. In this study, the t _½_ decreased by increasing the temperature. At 60 °C, the t _½_ was 136.5 min.

The *z* value was estimated from the slope of Fig. 5b. It was noticed that the *z* value of partially purified cellulase was 28.74 °C.

The activation energy of denaturation (E_d_) for thermal denaturation was 69.81 kJ/mol which was determined from first-order Arrhenius plot Fig. 5c. This value indicated that a high energy was necessary for thermal deactivation of crude cellulase enzyme. The E_d_ value was used to estimate the thermodynamic values of variation of enthalpy (ΔH_d_). The enthalpy is the quantity of heat needed to denature the enzyme. Positive ΔH_d_ values were found at the examined temperatures (Table 2), showing that the inactivation reaction is endothermic. At 40 °C, ΔG_d_ levels are greater than at 50 °C, 60 °C, and 70 °C.

### Discussion

Wheat bran was used as the main substrate in this study because the consumption of wheat bran's starch and hemicellulosic/cellulosic components would substantially simplify prospective biorefinery uses. So, it has the potential to be as a low-cost feedstock for the generation of renewable energy or chemicals (Yang and Wyman 2008). Wheat bran is the outer coat of wheat grain (*Triticum aestivum* L.) which comprises of the outer coat (pericarp, testa and aleuron layers). Milling separated it from the other portions of the wheat kernel.

*V. salarius* is a rod-shaped, moderately halophilic gram-positive, endospore-forming, bacterium that was isolated from a salty soil in Egypt. To our knowledge, little literatures discussed the capacity of *V. salarius* to produce cellulase enzyme and decompose cellulosic compounds. This bacterial species was firstly isolated from Gharsa Salt Lake (Chott el Gharsa), Tunisia as a novel *Virgibacillus* species (Hua et al. 2008). Other literatures mentioned the role of *Virgibacillus salaries* in bioremediation of medical wastes via production of hydrolytic enzymes and production of CMCase (Bhatt et al. 2018; Ethica and Sabdono 2021).

Cellulase yields are determined by a complicated interaction involving variables such as the producing bacteria, type of substrate, and incubation period (Acharya and Chaudhary 2012). It was noticed that cellulose exhibited low saccharification percentage which may be attributed to a complex structure, high crystallinity and polymerization degree of cellulose and water hydrophobicity. So, it hardly to be attacked by the bacterial enzymes as it is not available to their cells. On the other hand, the natural cellulosic wastes i.e., corn stover, sugarcane bagasse and wheat bran exhibited low polymerization that enable the fast utilization and variation of functional groups on their surfaces that facilitate the attachment of bacterial cells to their surfaces and increase the opportunity of utilization (Mussatto and Teixeira 2010; Teixeira et al. 2012). Wheat bran consists of nutrients rather than cellulose that may enhance the growth of bacteria and subsequently increase the induction of enzyme. Another explanation may suggest that the wheat bran and agricultural wastes may contain minerals that increase the activity of enzyme then the saccharification increased as previously mentioned by (Katileviciute et al. 2019). This interpretation come in context with the results of metal ion impacts the presence of metal ions enhanced the activity of enzymes.

The efficient lignocellulosic biomass utilization usually catalyzes by the synergistic process of three cellulolytic enzymes i.e. endoglucanase, cellobiohydrolase, and b-glucosidase (Chang and Wasser 2018; Porto de Souza Vandenberghe et al., 2022). The lower induction of cellulase towards cellulose meaning that the cellulase complex may include lower endoglucanase activity. Where, the cellobiohydrolase enzyme catalyze the hydrolysis of cellulose. In addition to, it has been reported that the higher microbial cellulase productivity is dependent upon the pretreatment of substrates compared with non-pretreated ones. These pretreatments would be acid and/or alkali, organic solvents, or steam explosion (Carrasco et al. 1994; Mahmoud et al. 2021; Zhong et al. 2015). Compared with different pretreatments, bacterial cultures were considered as an efficient pretreatment strategy without any chemical’s consumption or dramatic physical circumstances (such as high temperature, and high pressure) (Amarasekara and Shanbhag 2013). The saccharification percentage of the alkaline/acid pre-treated date palm wastes was effective higher than the untreated wastes (Alrumman 2016). Alkali pre-treatment of wheat straw, rice straw, and bagasse improved the enzymatic saccharification of cellulases by *Bacillus subtilis*, with saccharification rates of 33.0%, 25.5%, and 35.5%, respectively (Akhtar et al. 2017). However, in the current study, *V. salarius* strain BM02 produced cellulase on raw substrates without any prior treatments with relatively higher saccharification percentages compared the previously mentioned studies. This paved the way to use this strain as a promising candidate for cellulase bio-industrial application. The similar results were obtained from *Haloarcula* sp. LLSG7 (Li and Yu 2013). Moreover, (Meng et al. 2014) found that *B. subtilis* BY-3 produced cellulase enzyme when grown on various carbon sources with the maximum value on the corn stover. In this study, the presence of lignin in wheat bran did not show adverse impact on the activity of enzyme may be due to the bacteria produced a lignocellulosic enzymes that not only hydrolyze cellulose, but also it breaks down the lignin. In addition to when the wheat bran was chemically analyzed, the percentage of polyphenols (lignin) was 8.17% which was a significantly low compared with the percentage of total carbohydrate (64%).

It was noticed that the increasing of  substrate concentration (wheat bran) over 30 g/L followed by decreasing in cellulase activity and the saccharification percentages. This may be attributed to, cellulase inhibition by saccharification products and suppression in the synergetic action between cellulase enzymes complex as previously mentioned by (Hari Krishna and Chowdary 2000; Wen et al. 2004). This result agreed with the results obtained by (Ouyang et al. 2009) which reported that the concentration of 3% of corncob residue produced maximum saccharification yield (90%). Moreover, Alrumman (2016) reported that 4% of alkaline pretreated wheat straw was the best concentration for maximum saccharification yield.

The enzymes’ operation conditions i.e., pH, temperature, metal ions and salinity play a critical and control role in the activity and stability of them. Previous studies reported the activity of cellulase on different pH range. The cellulase of *Bacillus subtilis* BY-3 was most active at pH 5.5 (Meng et al. 2014). *Bacillus subtilisYJ1* cellulase was most active at pH 6.0–6.5 and stable between pH 6.5 and 7.5 (Yin et al. 2010). The optimal pH of cellulase activity of *Bacillus* strains was ranged from 5.0 to 6.5 (Mawadza et al. 2000). The optimum pH for cellulase extracted from *Rhizopus oryzae* PR to catalyze the hydrolysis of various agro-wastes pH 7.0 (John et al., 2007).

Regarding to the impact of salinity on the enzyme activity, it was found that, cellulase activity induced from *V. salarius* was NaCl concentration dependent. The same finding was reported by (Delgado-García et al. 2012). They discussed that a halotolerant strain *Salinivibrio* sp. depended on Na^+^ ion for achieving maximum hydrolase enzyme activity (5% of NaCl), however, the produced enzyme was active over a range of 1–15% of NaCl and bacterium was able to grow even in the absence of NaCl. Moreover, the optimal activity of *Paenibacillus tarimensis* L88 extremotolerant cellulases was evaluated at 80 °C and pH (3.0–10.5) in the presence of a high salt content (Raddadi et al. 2013). Variable enzymes, as cellulases, amylases, and ligninase that extracted from halophilic and halotolerant bacteria serve as potential industrial applications using renewable sources (Amoozegar et al. 2019; Elmansy et al. 2018; Schreck and Grunden 2014).

It was noticed that the application of metal ions exhibited a positive impact on the capacity of cellulase hydrolysis. This came in context with (Kamireddy et al. 2013; Wang et al. 2018). They mentioned that the addition of metal ions to lignocellulosic materials during pretreatment process enhanced the digestibility of enzymes. Addition of Mg^+2^ and NH_4_^+^ increased the xylan digestion (Kang et al. 2013), while addition of Fe^+2^ and Fe^+3^ to corn stalk enhanced saccharification yield and elimination of hemicellulose. The addition of Cu^+2^ and Fe^+3^ increased corn stalk enzymatic hydrolysis more than acid pretreated samples (Wang et al. 2018). (Yousef et al. 2019) reported that calcium nanoparticles exhibited enhancement of cellulase activity by endophytic bacterial strain. In the current study, the significant (*p* ≤ *0.05*) increase in the cellulase rate when Fe^+3^ was added may be owing to Fe^+3^ served as a cellulase activator that altered the structure of the cellulase protein, increased the quantity of active enzymes while decreased non-productive enzyme adsorption. As a result, the activity of the cellulase enzyme enhanced. These results agreed with (Wang et al. 2018) who mentioned that the Fe^+3^ ion exhibited as a canal that connected between active site cellulase and its substrate. So that, it accelerated the reaction and enhanced hydrolytic activity of enzyme. EDTA is a metal ion chelating agent, therefore it may chelate metal ion cofactors and then inactivate cellulase enzyme. That’s why the addition of EDTA inhibit the activity of enzymes and it suggested that cellulase enzyme activity is metal cofactor dependent (Elbanna et al. 2015). The inhibitory impact of ionic surfactant SDS is attributed to the distortion in the protein active site by non-specific interactions, causing conformational changes which result in protein unfolding and instability, the same finding was documented by (Malik and Javed 2021).

Regrading to enzyme kinetics, a lower Km value indicates that an enzyme has a strong affinity for its substrate. It is the quantity of substrate necessary to execute half of the maximum beginning velocity (Tong et al. 1980). A cellulase with Km value ranged between 0.6 and 7.2 mg| mL for CMC has been reported by Wang et al. (2018). A Lineweaver–Burk double reciprocal plot was used to calculate the kinetic characteristics of crude cellulase, which had a Km value of 5.3 mg/mL and a Vmax value of 7.28 U/mL (Saqib et al. 2012).

The high optimum temperature range for optimum enzymes activity as well as the high stabilities at high temperature are critical factors when applying the enzymes in the industrial processes particularly, the temperature above 50 °C (Bhatti et al. 2007; Yeoman et al. 2010). At 60 °C, the t_½_ was 136.5 min that was considered long compared to t _½_ value was free and immobilized cellulase enzyme; 22 and 30 min, respectively which was reported by (Karim et al. 2021). Moreover, the higher the *z* value of an enzyme, the more its sensitivity to the time of thermal treatment, and the lower the *z* value, the more its sensitivity to temperature increases (Tayefi-Nasrabadi and Asadpour 2008).

The enthalpy ΔH_d_ values are rather high, suggesting that the enzyme is thermally stable. The breakdown of non-covalent connections, particularly hydrophobic contacts, in the enzyme structure is connected with a modest decrease in enthalpy with increasing temperature (Daniel 1996). Lower ΔH_d_ at higher processing temperatures indicated that cellulase thermal inactivation was simpler. The change in Gibbs free energy (ΔG_d_) is a more accurate indicator of enzyme thermostability. The high ΔG_d_ values showed a high degree of thermostability. When enzymes are thermally denaturized, the opening of the enzyme structure is followed with an increase in disorder or entropy (ΔS_d_), and hence positive values of ΔS_d_ were recorded (Rashid and Siddiqui 1998; Siddiqui 2017). Since the ΔS_d_ values were negative (Table 2), the native form of cellulase is more in ordered state. Moreover, it suggested that there is no significant aggregation of protein during denaturation (Agrawal et al. 2019). The negative value for ΔS_d_ in the current study explained that the entropy reduced in forming the transition state. A low ΔS_d_ value signifies the existence of the enzyme in its stable state (D'Amico et al. 2003). When ΔH_d_ is positive and ΔS_d_ is negative, the process is not spontaneous at any temperature. Therefore, inactivation of cellulase can be reversible between 40 and 70 °C as was reported by (Saqib et al. 2012).

Thermophilic bacteria are frequently regarded as a source of industrially important thermostable enzymes (Rigoldi et al. 2018). An enzyme is thermally stable if it has a high specified unfolding (transition) temperature (Tm) or a lengthy half-life at a high temperature (Böhme et al. 2020). These distinct properties of thermostable enzymes open the door for their broad use in industry. Because of their high catalytic activity and capacity to tolerate the heat generated in many bio-industrial processes, thermostable enzymes derived from thermophilic bacilli, for example, have found a myriad of commercial uses (Margaryan et al. 2018). As a result, thermophilic enzymes can be utilized to catalyze high-temperature chemical reactions that are difficult for normal-temperature enzymes to catalyze. It has reported that the halotolerant bacterial species have an outstanding endoglucanase enzymes stability compared with other ordinary species (Zhu et al. 2020). (Li and Yu 2013) isolated *Haloarcula* sp. Strain LLSG7 from Yuncheng Salt Lake, China with higher crude cellulase stability and cellulolytic activity in the presence of organic solvents.

### Conclusion

*Virgibacillus salaries* BM02 could utilize many agro-wastes as substrates with high saccharification yield without any prior treatment processes. wheat bran was the best substrate that stimulate high cellulase induction. The produced cellulase enzyme exhibited halo-thermo stability. It can be active and stable at 12% NaCl and 60 °C. Therefore, this strain is recommended to be used as a candidate for many applications such as (i) production of bio-industrial cellulase with a reliable, thermostable, and halotolerant activity, (ii) no need to pretreatment of substrates to produce enzyme where this step is cost effectiveness and safe a time for commercial cellulase production, (iii) utilization of lignocellulosic wastes. Because of *V. salaries* BM02 performed in extremophilic conditions such as high temperature and high salt concentration, it encourages the introducing it in the industrial application via simultaneous saccharification and co-fermentation (SSCF) simultaneous saccharification and fermentation (SSF) processes.

## Data Availability

The author confirm that the data of the current study are available from the corresponding author on reasonable request.
